# PERCEIVED SOCIOECONOMIC STATUS, PRODUCTIVE SOCIAL ENGAGEMENT, AND SUBJECTIVE HEALTH: LONGITUDINAL STUDY IN TAIWAN

**DOI:** 10.1093/geroni/igad104.3081

**Published:** 2023-12-21

**Authors:** Chin-Yi Su, Shiau Fang Chao

**Affiliations:** Taiwan Association of Family Caregivers, Taipei, Taipei, Taiwan (Republic of China); National Taiwan University, Taipei City, Taipei, Taiwan (Republic of China)

## Abstract

Productive social engagement (PSE) has been identified as a crucial factor in promoting subjective health in later life. However, there has been limited focus on how perceived socioeconomic status (SES) among older adults mediates this relationship over the long term. This study employed data from the Taiwan Longitudinal Study on Aging to examine a sample of 2,865 individuals aged 55 or above from 2011 to 2015. By employing a path analysis, this study tested the hypothesized effects developed from active aging theory and social capital theory. Results indicates that : 1. Higher levels of PSE and perceived SES predicted higher subjective health within the same wave, both in T1 and T2. 2. Perceived SES mediated the relationship between PSE and subjective health only in T1. Also, the effect of T1 PSE on T2 PSE operated through T1perceived SES. 3. T1 PSE positively predicted T2 subjective health through T1 perceived SES and T2 perceived SES over the time. This study sheds light on the mechanism linking PSE, perceived SES, and subjective health. The findings suggest that higher perceived SES is associated with more PSE in a four-year interval, which in turn predicts higher subjective health. This indicates that social capital may facilitate older adults’ access to PSE over time. Future research should investigate the role of perceived SES and how it interacts with objective and subjective physical well-being in later life. Interventions should target ways to help older adults access opportunities for engaging in productive activities to improve their health.

